# Classification of ductal carcinoma *in situ *by gene expression profiling

**DOI:** 10.1186/bcr1613

**Published:** 2006-10-30

**Authors:** Juliane Hannemann, Arno Velds, Johannes BG Halfwerk, Bas Kreike, Johannes L Peterse, Marc J van de Vijver

**Affiliations:** 1 Division of Experimental Therapy, Netherlands Cancer Institute, Plesmanlaan 121, 1066 CX Amsterdam, The Netherlands; 2 Central Microarray Facility, Netherlands Cancer Institute, Plesmanlaan 121, 1066 CX Amsterdam, The Netherlands; 3 Division of Radiotherapy, Netherlands Cancer Institute, Plesmanlaan 121, 1066 CX Amsterdam, The Netherlands; 4 Division of Diagnostic Oncology, Netherlands Cancer Institute, Plesmanlaan 121, 1066 CX Amsterdam, The Netherlands

## Abstract

**Introduction:**

Ductal carcinoma *in situ *(DCIS) is characterised by the intraductal proliferation of malignant epithelial cells. Several histological classification systems have been developed, but assessing the histological type/grade of DCIS lesions is still challenging, making treatment decisions based on these features difficult. To obtain insight in the molecular basis of the development of different types of DCIS and its progression to invasive breast cancer, we have studied differences in gene expression between different types of DCIS and between DCIS and invasive breast carcinomas.

**Methods:**

Gene expression profiling using microarray analysis has been performed on 40 *in situ *and 40 invasive breast cancer cases.

**Results:**

DCIS cases were classified as well- (*n *= 6), intermediately (*n *= 18), and poorly (*n *= 14) differentiated type. Of the 40 invasive breast cancer samples, five samples were grade I, 11 samples were grade II, and 24 samples were grade III. Using two-dimensional hierarchical clustering, the basal-like type, ERB-B2 type, and the luminal-type tumours originally described for invasive breast cancer could also be identified in DCIS.

**Conclusion:**

Using supervised classification, we identified a gene expression classifier of 35 genes, which differed between DCIS and invasive breast cancer; a classifier of 43 genes could be identified separating between well- and poorly differentiated DCIS samples.

## Introduction

Ductal carcinoma *in situ *(DCIS) of the breast represents a heterogeneous group of non-invasive breast tumours commonly detected in women undergoing screening mammography. DCIS is characterised by malignant epithelial cells accumulating in the ducts of the breast without invading through the basement membrane into the surrounding tissue. DCIS accounts for approximately 3% of symptomatic breast malignancies and for approximately 20% of breast malignancies in patients from population-based screening programs [1].

Different histological types of DCIS can be recognised, and a variety of classification systems have been developed [2]. Due to subjective interpretation of the morphology of the lesions, even experienced pathologists differ in their classification of DCIS [3]. Therefore, histological classification of DCIS may not be sufficient, and additional classification approaches could assist pathological classification.

It is assumed that most cases of DCIS will progress to invasive breast cancer. Because this progression may take many years and may not occur within the lifetime of a patient, elucidating the mechanisms of progression from *in situ *lesions to invasive disease and developing diagnostic tests would be of great clinical benefit.

Several models of the evolution of DCIS to invasive cancer have been suggested. One model suggests the linear progression from low-nuclear-grade DCIS to high-nuclear DCIS and the subsequent development of invasive cancer [4]. Based on specific genetic alterations found in the different types of DCIS, a more likely scenario is the evolution of well-, moderately, and poorly differentiated DCIS via distinct pathways. Following this idea, well-differentiated DCIS can give rise to low-grade invasive carcinoma, whereas poorly differentiated DCIS can give rise to high-grade invasive breast cancer [5,6].

Several specific genetic alterations have been found in DCIS. HER2 gene amplification and protein overexpression are detected in up to 70% of poorly differentiated DCIS cases [7], and cyclin D1 is amplified and overexpressed in DCIS [8] in approximately 20% of the cases. Inactivating mutations of the E-cadherin gene are detected in almost all cases of lobular carcinoma *in situ *(LCIS) [9]. Several other genetic alterations in oncogenes (for example, C-MYC) and tumour suppressor genes (for example, p53) have been found in DCIS and are reviewed in Reis-Filho and colleagues [10] and Allred and colleagues [11].

Gene expression profiling has been shown to be a powerful tool for identifying profiles of tumour subtypes [12-15] and for correlating gene expression profiles with outcome in breast cancer [16-18]. The identification of specific gene expression patterns correlated with the different types of DCIS may help to elucidate the processes underlying the evolution of *in situ *carcinomas of the breast and also lead to a more reproducible classification of DCIS lesions.

To date, only a few studies of gene expression profiling of DCIS and a comparison with the gene expression pattern of invasive samples have been published and these are based on a small number of samples [19,20].

In the study presented here, gene expression profiling was performed on one LCIS and 39 DCIS samples to identify differentially expressed genes between well-, intermediately, and poorly differentiated DCIS. In addition, differences in gene expression between these cases of carcinoma *in situ *and 40 invasive breast carcinomas were studied.

## Materials and methods

### Selection of samples

Cases of DCIS were selected from the tissue bank of the Netherlands Cancer Institute (Amsterdam, The Netherlands). These samples were obtained within 1 hour after surgery from patients who underwent wide local excision (*n *= 16) or mastectomy (*n *= 24). All samples were reviewed by two pathologists independently to determine the histological classification of the samples according to Holland and colleagues [21]; samples were classified as well, intermediately, or poorly differentiated. For analysis purposes, the intermediately differentiated DCIS cases were subclassified as those cases that were in part well differentiated (well to intermediately differentiated) and those that were in part poorly differentiated (moderately to poorly differentiated) in some areas. In cases in which there was a discrepancy in classification between the two pathologists, the histological slides were reviewed together to reach an agreement.

In addition, 40 cases of primary invasive breast cancer were selected; these were all cases of invasive ductal carcinoma (IDC) measuring between 1 and 5 cm and were graded as grade 1, 2, or 3 according to the method described by Elston and Ellis [22]. The study was approved by the medical ethical committee of the Netherlands Cancer Institute.

### RNA isolation and amplification

RNA isolation and amplification were performed essentially as described by Weigelt and colleagues [23]. Thirty tissue sections of 30 μm of frozen material were cut. The first and the last tissue sections were 6 μm in thickness and were stained with haematoxylin and eosin to determine the percentage of tumour cells and to exclude invasive growth. Only samples with greater than or equal to50% of tumour cells were used for gene expression profiling.

### Immunohistochemistry

The procedures applied are described in the supplementary information provided online [24].

### Microarray hybridisation

Labeling of the amplified cRNA and microarray hybridisations were performed as previously described [25]. Equal amounts of amplified cRNAs of 100 invasive breast carcinomas were pooled and used as a reference. All hybridisations were performed on 18K human cDNA arrays (Central Microarray Facility, Netherlands Cancer Institute) [26].

Microarrays were scanned with the DNA Microarray Scanner G2565B (Agilent Technologies, Santa Clara, CA, USA). Self-self hybridisations were used to validate the quality of the hybridisations and as a negative control in the error model.

### Processing of microarray data

Information on data processing is provided in the supplementary information [24].

### Unsupervised hierarchical clustering

Two-dimensional unsupervised hierarchical clustering using Pearson correlation as distance function and complete linkage was performed using Genesis software (Technical University, Graz, Austria) [27,28].

### Supervised classification

We performed supervised classification applying methods described previously [16,29,30]. Pathological features (histological type of the DCIS samples, histological grade of the invasive samples) were used to define groups for supervised classification. Genes were rank-ordered based on their signal-to-noise statistic. Safe cutoffs were determined by comparing the signal-to-noise ratio (SNR) values with the results from 2,000 sample label permutations (Monte Carlo randomisation). For each group and a number of genes, a centroid is defined as the mean ratio per gene over all samples in that group. Correlation or Euclidean distance of each sample to those centroids determines their predicted group. Leave-out cross-validation was used to determine the optimal number of genes separating the groups. The number of left-out samples in this cross-validation procedure was dependent on the number of samples within the analysis set. SNR calculation, Monte Carlo randomisation, and cross-validation have been described previously [25].

### Supplementary information

The microarray data, additional information on the methods, and the filtering results are provided as supplementary information [24].

## Results

This study was performed to identify differences in gene expression (a) between DCIS and invasive breast cancer and (b) between different histological types of DCIS.

### Tumour characteristics

Thirty-nine cases of DCIS of the breast were included in the analyses. By histological examination, they were assigned to the following groups: well differentiated (*n *= 6), intermediately differentiated (*n *= 18), and poorly differentiated (*n *= 14). For analysis purposes, the group of intermediately differentiated cases was further subdivided in well-intermediately (*n *= 10), true intermediately (*n *= 2), and intermediately-poorly (*n *= 6) differentiated type. One sample contains a mixture of well- and poorly differentiated DCIS components in the same tissue specimen. In addition, one case of LCIS was included.

To be able to compare DCIS with invasive breast cancer, 40 cases of invasive breast cancer were studied. Five tumours were histological grade 1, 11 samples were grade 2, and 24 samples were grade 3. Patient and tumour characteristics are summarised in Table 1.

### Molecular subtypes of breast cancer

Several subtypes of breast cancer have been identified by gene expression profiling and have been correlated with clinical outcome [13,14]. This classification has been translated to classical immunohistochemistry (IHC): basal-type tumours are characterised by negative staining for oestrogen receptor (ER), progesterone receptor, and HER2 and are often positive for keratin 5/6; ERB-B2 tumours are HER2-positive, and luminal A and B tumours are ER-positive and HER2-negative. In our set of 40 *in situ *tumours, only two tumours are positive for CK5/6 by IHC. Both of them are poorly differentiated and negative for HER2 and ER by IHC. From the intrinsic gene set identified by Perou and colleagues [12], we could match 403 identifiers to our array platform. This set of genes was used to perform unsupervised hierarchical clustering of the 40 *in situ *samples. We clearly see a discrimination between tumours highly expressing genes of the luminal/ESR1 cluster and tumours negative for these genes, whereas the discrimination for the HER2-overexpressing groups was much less clear (Figure 1 in the supplementary information [24]). We could not identify a large basal-type group, which is in agreement with the data obtained using IHC.

### Unsupervised hierarchical clustering

#### Unsupervised hierarchical clustering of *in situ *and invasive samples

**Figure 1:**
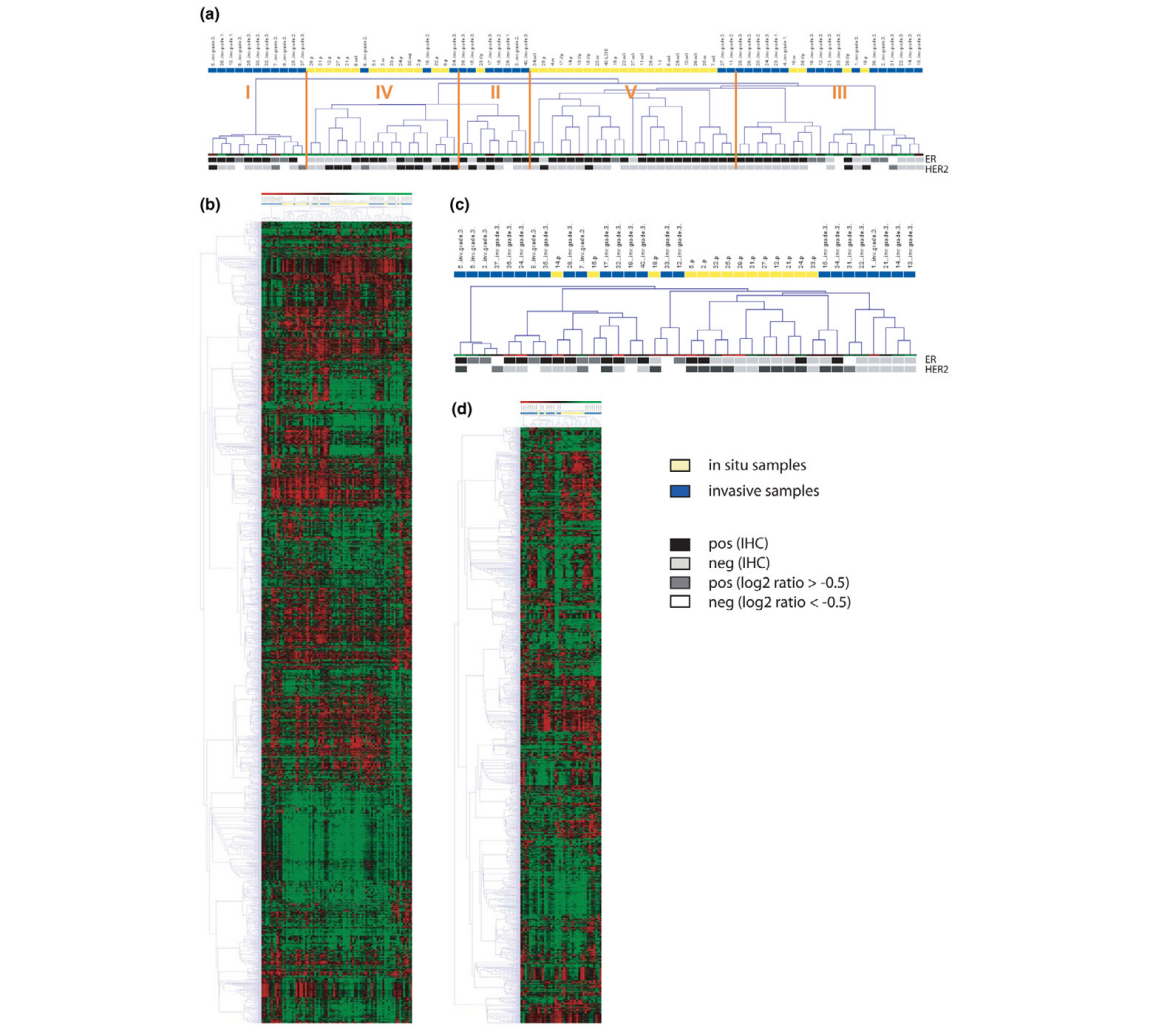
Unsupervised hierarchical clustering of *in situ *and invasive samples. **(a) **Dendrogram of all *in situ *(*n *= 40) and all invasive (*n *= 40) samples. **(b) **Scaled-down representation of the entire cluster shown in **(a) **(1,706 genes). **(c) **Dendogram of poorly differentiated ductal carcinoma *in situ *(*n *= 14) and grade 3 invasive (*n *= 24) samples. **(d) **Entire cluster of **(c) **(1,119 genes). Yellow indicates *in situ *samples, and blue indicates invasive samples. i, intermediately differentiated; IHC, immunohistochemistry; i/p, intermediately/poorly differentiated; LCIS, lobular carcinoma *in situ*; p, poorly differentiated; w, well differentiated; w/i, well/intermediately differentiated.

First, the whole group of DCIS and invasive samples was clustered (Figure 1a). As can be seen, the invasive samples cluster in three different groups (indicated as I, II, and III in Figure 1a). Ten out of 14 poorly differentiated DCIS samples cluster together in a fourth group, and a fifth group consists of 13 out of 18 cases of intermediately differentiated DCIS and four out of six of the well-differentiated *in situ *samples. The clustering seems not to be driven mainly by the ER status or the HER2 status of the samples. These results suggest that poorly differentiated DCIS samples show an overall gene expression profile other than that of the intermediately and well-differentiated DCIS samples.

#### Unsupervised hierarchical clustering of DCIS

We also performed unsupervised hierarchical cluster analysis to the series of DCIS cases only, resulting in two large groups. One group contains 10 poorly differentiated samples and only one well-differentiated sample, whereas 83% of the well-differentiated samples group in the other, second cluster. Most of the samples in this second group are ER-positive by IHC. In total, our sample set contains 18 cases with an intermediately differentiated component. Of these samples, 12 cluster in the arm of the well-differentiated samples. In accordance with the clustering results presented in Figure 1, these results also indicate that the overall gene expression profiles of *in situ *samples with an intermediately differentiated component are more similar to those of well-differentiated DCIS than to those of poorly differentiated DCIS. It is clear from these results that there are large differences in gene expression pattern between well- and poorly differentiated DCIS.

### Supervised classification

We performed supervised classification on different data sets to identify the genes differentially expressed between the groups of interest. These groups are (a) 40 *in situ *versus 40 invasive breast carcinomas, (b) 14 poorly differentiated DCIS cases versus 38 invasive grade 3 tumours, and (c) six cases of well-versus 14 cases of poorly differentiated DCIS.

#### Supervised classification of *in situ *versus invasive carcinomas

We investigated the differences in gene expression between *in situ *and invasive breast carcinoma samples. We therefore used the whole data set and assigned all 40 *in situ* samples to one group and all 40 invasive samples to a second group (analysis set 1). To obtain a profile taking into account the expression sets of both tumour types, significantly regulated genes were identified independently for both groups. The 1,706 overlapping genes were used for analysis. Monte Carlo randomisation revealed approximately 300 genes differentially expressed between *in situ *and invasive samples.

After cross-validation, classifier consisting of 35 genes resulted in a stable prediction of the differences between DCIS and invasive breast carcinomas, with an average performance of 91%. The gene list is provided in Table 2.

#### Supervised classification for poorly differentiated DCIS versus grade 3 invasive carcinoma

**Figure 2:**
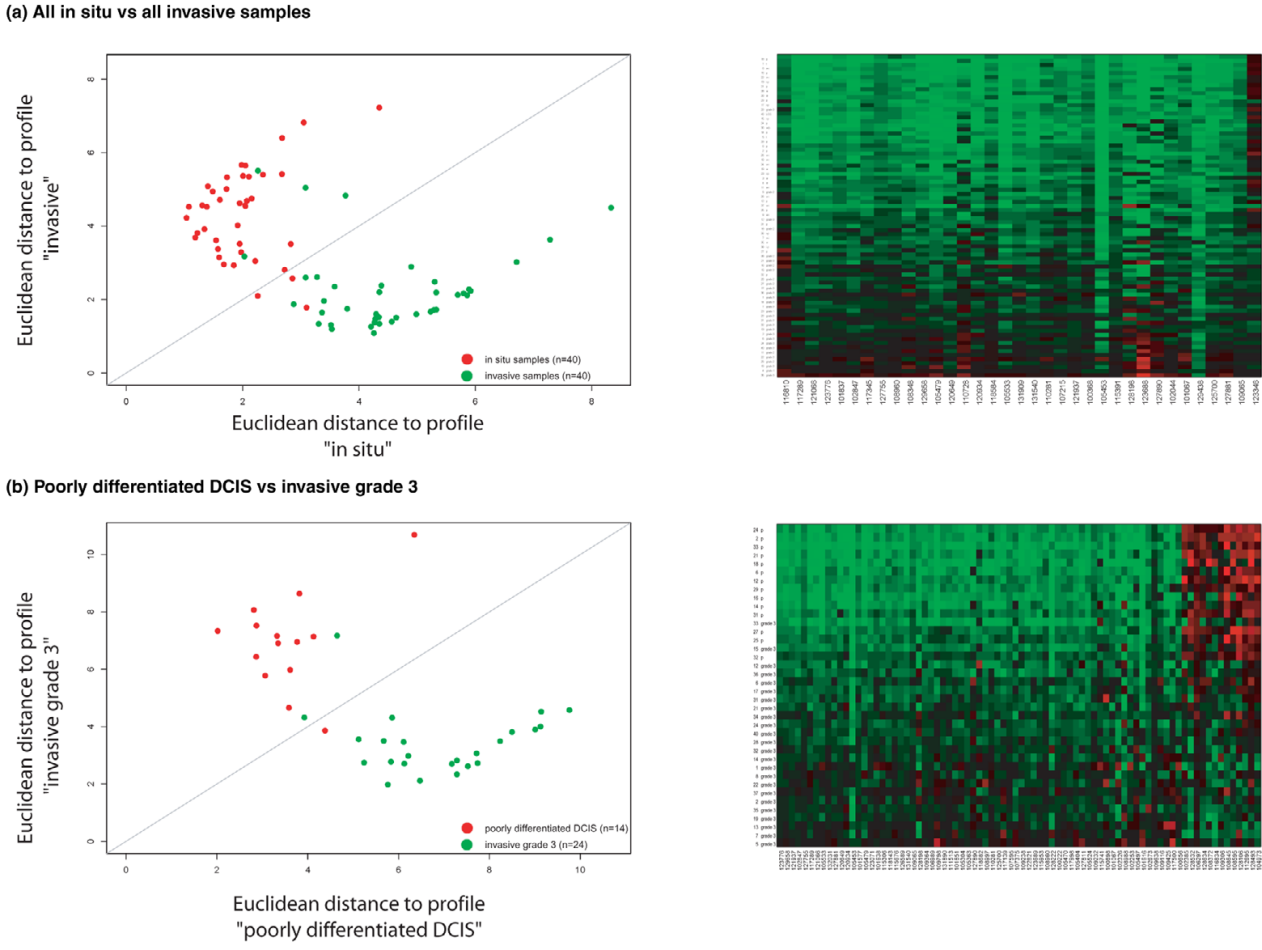
Euclidean distance and heatmaps of the *in situ *and invasive samples using the classifiers obtained after cross-validation. **(a) **All ductal carcinoma *in situ *(DCIS) (*n *= 40) and all invasive (*n *= 40) samples. The classifiers consist of 80 genes. **(b) **Poorly differentiated DCIS (*n *= 14) versus invasive grade 3 samples (*n *= 24) using a classifier of 35 genes. p, poorly differentiated.

Because it is very likely that grade 3 invasive breast cancer arises from poorly differentiated DCIS [5,6], we applied the supervised classification procedure to the subset of poorly differentiated DCIS (*n *= 14) and grade 3 invasive tumours (*n *= 24) (analysis set 2). Again, the filtering procedure was applied to both groups independently. The overlapping fraction of this gene list contains 1,119 genes that were used to perform the analyses. Monte Carlo randomisation showed that 80 genes are differentially expressed between poorly differentiated DCIS and grade 3 invasive breast carcinoma samples. After cross-validation in 14 steps, the best performance of 93% is reached, when at least 50 genes are used to build the classifier. This performance remains stable with increasing numbers of genes. This means that 50 to 80 genes are able to discriminate between poorly differentiated DCIS and invasive grade 3 breast tumours (Figure 2a). These 80 genes are shown in Table 3. Between the 35-gene classifier of all DCIS and invasive samples and the subgroup classifier of 80 genes, 21 genes were present in both classifiers.

#### Supervised classification of well-versus poorly differentiated DCIS

**Figure 3:**
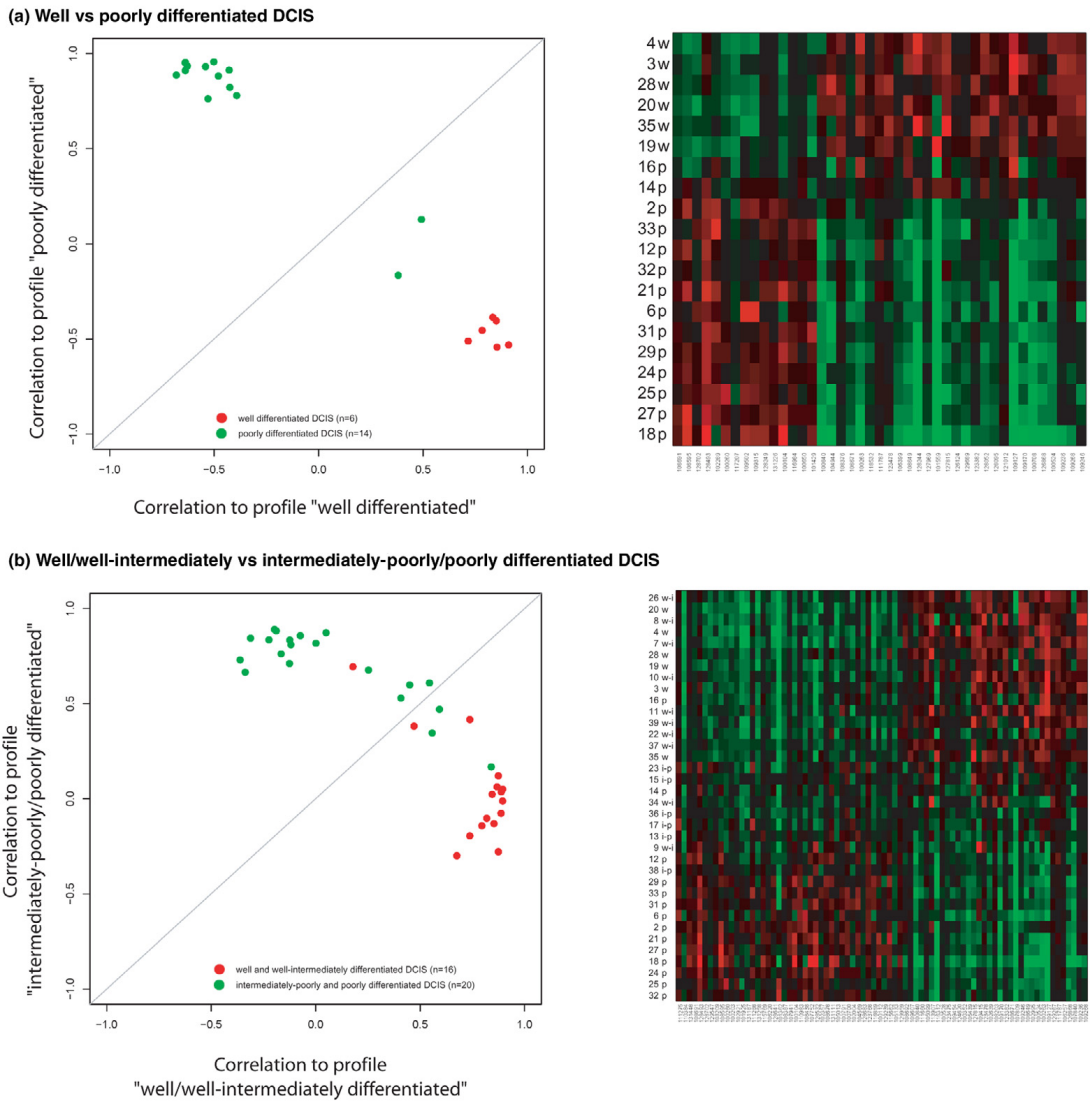
Correlation plots and heatmaps of the *in situ *samples using the classifiers obtained after cross-validation. **(a) **Well- (*n *= 6) versus poorly (*n *= 14) differentiated ductal carcinoma *in situ *(DCIS). The classifiers consist of 43 genes. **(b) **Well-/well-intermediately (*n *= 16) versus intermediately-poorly/poorly (*n *= 20) differentiated DCIS using a classifier of 78 genes. i-p, intermediately-poorly differentiated; p, poorly differentiated; w, well differentiated; w-i, well-intermediately differentiated.

We intended to find the most prominent differences between the well- and poorly differentiated DCIS samples. Sixfold cross-validation of six well- and 14 poorly differentiated *in situ *samples (analysis set 3) resulted in a set of 43 genes separating these groups with a performance of 90% (Figure 3a, Table 4).

Because histological classification of intermediately differentiated DCIS versus well- or moderately differentiated DCIS is most challenging, we investigated whether gene expression profiling could be used to identify markers that could help in making this classification. We therefore included the cases classified as intermediately differentiated DCIS. Subsequently, we divided the sample set into one group of well/well-intermediately differentiated samples (*n *= 16) and a second group containing poorly/intermediately-poorly differentiated samples (*n *= 20). Supervised classification of these data revealed a set of 78 genes separating these two groups with an average performance of 89% (Table 5).

**Figure 4:**
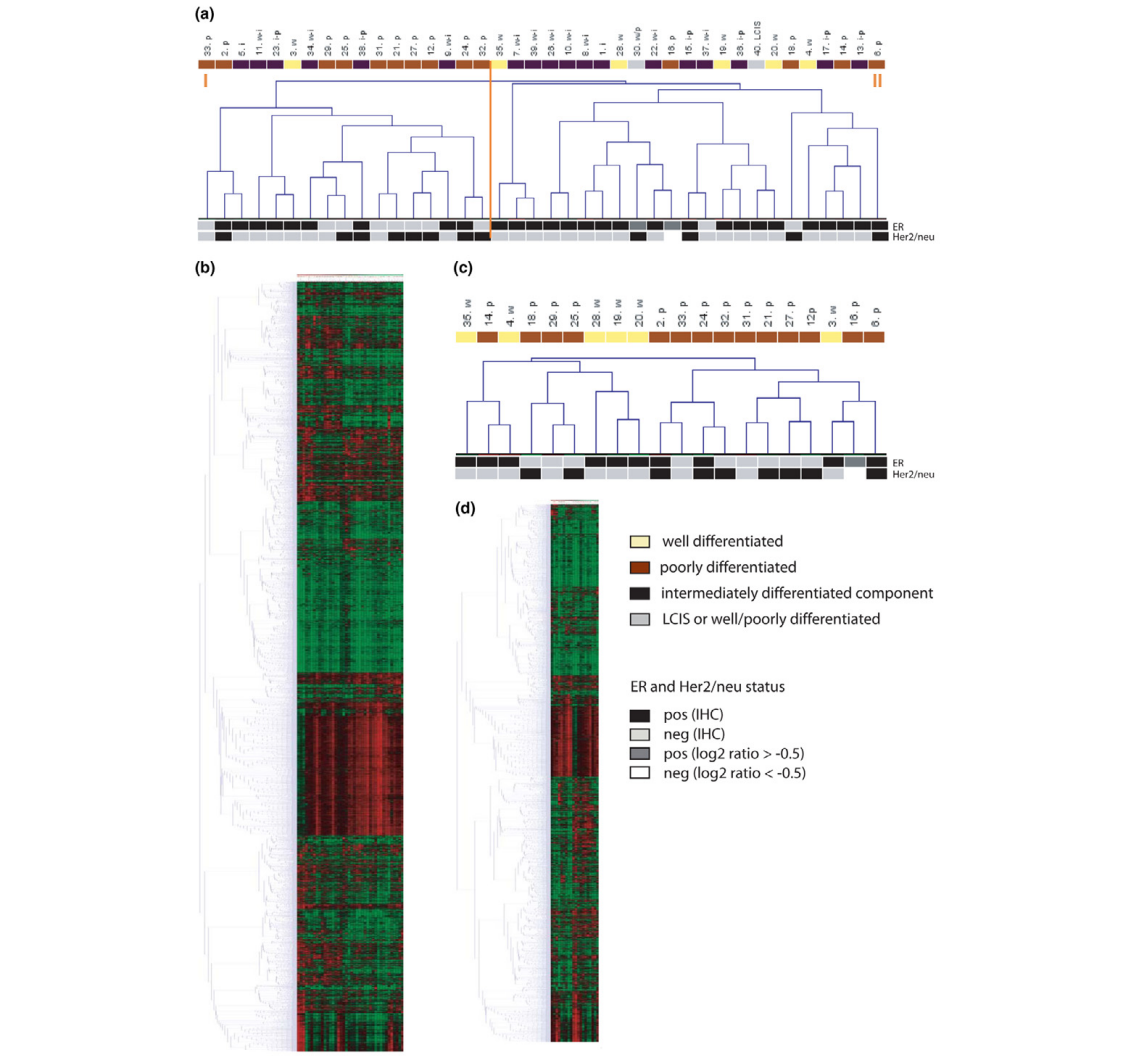
Unsupervised hierarchical clustering of the *in situ *samples. **(a) **Dendrogram of all 40 *in situ *samples. **(b) **Scaled-down representation of the entire cluster of **(a) **(5,788 genes). **(c) **Dendrogram of only the well- (*n *= 6) and poorly (*n *= 14) differentiated ductal carcinoma *in situ *(DCIS) cases. **(d) **Entire cluster of **(c) **(4,493 genes). Yellow indicates well-differentiated DCIS, brown indicates poorly differentiated DCIS, black indicates DCIS samples with an intermediately differentiated component, and gray indicates special cases. i, intermediately differentiated; IHC, immunohistochemistry; i-p, intermediately-poorly differentiated; LCIS, lobular carcinoma *in situ*; p, poorly differentiated; w, well differentiated; w-i, well-intermediately differentiated.

We observed a separation of this data set in three distinct groups (Figure 3). One group contains one intermediately-poorly differentiated sample (17%) and 12 out of 14 poorly differentiated samples, and a second group all six well-differentiated samples and seven out of 10 well-intermediately differentiated samples. The third group shows no correlation with both profiles and consists of five out of six intermediately-poorly and three out of 10 well-intermediately differentiated samples. This implies that this third group typifies mainly the intermediately-poorly differentiated samples. Well-intermediately differentiated samples are apparently very similar to well-differentiated DCIS in their gene expression. These results are in accordance with the results of unsupervised hierarchical clustering of all *in situ *samples (Figure 4a).

Twenty-one genes are overlapping between the 43 genes of analysis set 3 and the 78 genes of analysis set 4. It is known that many poorly differentiated *in situ *breast carcinomas do not express the ER. In our data set, nine of all 14 poorly differentiated DCIS samples (64%) are negative for ER expression by IHC. There was a slight chance that our classifier would detect mainly the differences of ER-associated genes. We identified only one gene (*LIV-1*), beside the ER itself, directly ER-regulated in the classifier of 43 genes. Additionally, we compared the 43 genes with 2,460 ER-associated genes identified by van 't Veer and colleagues [16]. Thirteen genes, including the ER itself, have been found in both gene lists. So, most of the genes in this 43-gene classifier have not been correlated to ER expression so far, indicating that the differences between well- and poorly differentiated DCIS samples are not originating from the ER status of the samples.

Remarkably, completely different gene lists are found describing the differences in gene expression between different *in situ *samples, on one hand, and DCIS and invasive samples on the other hand. These findings may indicate that gene regulation involved in progression from *in situ *to invasive breast cancer affects molecular mechanisms other than the mechanisms responsible for the development of the different types of DCIS.

## Discussion

Although studies to identify gene expression signatures in DCIS are limited by difficulties in obtaining frozen material from DCIS, we were able to collect a relatively large series of DCIS cases for this purpose. It should be kept in mind that we did not have a sufficient number of cases to validate the gene expression signatures that we identified.

We were able to show that well- (*n *= 6) and poorly (*n *= 14) differentiated DCIS show different gene expression profiles and can be distinguished by a classifier of 43 genes. Most of the genes differentially expressed between well- and poorly differentiated DCIS are involved in metabolism (for example, *BTD*, *ETFA*, *GMFG*, and *PLAT*) and cell communication (for example, *ESR1*, *ACK1*, *CELSR2*, and *CCL19*).

One of the top genes in the 43-gene classifier is *BCL2*. The mRNA expression of this anti-apoptotic protein is upregulated in the well-differentiated samples. In addition to its anti-apoptotic function, BCL2 has a suggested role in neuro-endocrine differentiation in colon carcinomas [31] and its downregulation is associated with poor prognosis in breast cancer [32].

Twenty-eight of the 43 genes are upregulated and 15 genes are downregulated in the well-differentiated samples (Figure 3a). Whereas a number of the 28 upregulated genes are involved in DNA binding, no genes fulfilling this function are on the list of the 15 downregulated genes. Conversely, genes involved in phosphate metabolism (for example, *GMFG*, *ACK1*, and *ATP5B*) can be found within the 15 downregulated, but not in the 28 upregulated, genes.

It is known that HER2 is overexpressed in poorly differentiated DCIS in approximately 42% of the cases [7], and it has been suggested that HER2 overexpression is an early step in the evolution of a distinct type of breast carcinoma. In our data set of all *in situ *samples, we found a positive log2-ratio for HER mRNA expression in six of 14 poorly differentiated DCIS cases (43%) and in one case of intermediately-poorly differentiated DCIS. In all the other *in situ *samples, the log2-ratios of HER2 are negative. These results are in agreement with the hypothesis that HER2 overexpression is an early event in the development of poorly differentiated *in situ *breast carcinomas.

Supervised classification of well-, well-intermediately, intermediately-poorly, and poorly differentiated DCIS samples (analysis set 4) showed a separation of these samples in three groups: a 'good' group, a 'poor' group, and an 'intermediate' group containing mostly samples that were identified as intermediately-poorly differentiated samples by pathologists. This group also contains some samples pathologically classified as well-intermediately differentiated, whereas most of these samples fall in the 'good' group. These results indicate that well- and well-intermediately differentiated DCIS are more similar to each other than poorly and intermediately-poorly differentiated DCIS are. Following this idea, well- and well-intermediately differentiated samples may be considered to be one group, whereas poorly and intermediately-poorly differentiated samples seem to be two distinct groups of DCIS. If these results can be validated in additional studies, this classification could help to decrease controversial classification of DCIS due to interobserver variability and to recognise well-differentiated DCIS with more accuracy.

Within the gene lists describing the differences between well- and poorly differentiated DCIS, a number of genes refer to proteins for which antibodies are available. There is no single gene discriminating between the different types of DCIS, but it has to be investigated whether a combination of protein stainings in a patient's tissue can assist in better classification of DCIS. From the study presented here, potential candidates for such an approach are *Bcl-2*, *Ack1*, *CCL19*, and *CELSR2*, among others.

Thirty-five genes are able to describe the global differences in gene expression between *in situ *and invasive breast tumour samples. This classifier contains many genes involved in signal transduction (for example, *APC2*, *DAPK3*, *ADM*, *ARF1*, and *IQGAP1*) and cell growth and maintenance (*TGFB2*, *PTMS*, *PSAP*, *TUBB2*, and *MAP7*).

The most likely model describing the progression from *in situ *to invasive breast cancer lesions is the existence of distinct pathways for the evolution of well- and poorly differentiated DCIS. Following this idea, well-differentiated *in situ *lesions develop into grade 1 IDC, whereas poorly differentiated samples develop into grade 3 IDC [5,6]. We therefore performed supervised classification on the set of poorly differentiated DCIS (*n *= 14) and grade 3 invasive breast cancer (*n *= 24).

Approximately 80 genes discriminate poorly differentiated *in situ *from grade 3 invasive breast carcinomas. Thirteen of these 80 genes are upregulated and 67 genes are downregulated in poorly differentiated DCIS samples. The genes in this classifier are involved mostly in cell growth and protein metabolism. Many of them have a function in protein binding (for example, *LCP1*, *TRAP1*, *ID4*, *TOB1*, and *CDH*) and nucleic acid binding (for example, *FBL*, *PIAS4*, *ELF3*, *EIF4G1*, *NBS1*, and *WHSC1L1*).

A limited number of previous studies have addressed gene expression profiles in DCIS, and most of these studies have analysed a small number of samples. One study by Seth and colleagues [20] compared one case of low- to intermediate-grade DCIS with one case of high-grade DCIS with an invasive component and identified genes upregulated or downregulated in the low- to intermediate-grade DCIS case. Adeyinka and colleagues [19] studied six cases of DCIS with necrosis and four samples of DCIS without necrosis and identified a signature of 69 transcripts differentially expressed between these two groups. Ma and colleagues [33] used laser capture microdissection from paraffin-embedded material followed by gene expression profiling to identify molecular signatures in premalignant, preinvasive, and invasive stages of breast cancer. The results of their study suggested that tumour grade, rather than tumour stage, is associated with distinct gene expression patterns and that changes in gene expression required for invasive growth are already present in the DCIS stage [33]. In the study presented here, we compared the gene expression profiles of poorly differentiated DCIS lesions with those in grade 3 invasive breast tumours. In contrast to Ma and colleagues, we did not compare paired samples from the same patient but compared two groups of tumours. The 80-gene signature we identified is different from the signatures describing the differences between different grades of DCIS lesions. Schuetz and colleagues [34] identified gene expression signatures of *in situ *and invasive breast cancer by using 18 paired samples and combining laser capture microdissection and gene expression profiling on oligonucleotide microarrays. They showed that 546 probes were differentially expressed between DCIS and IDC. From the 18 genes they validated by real-time polymerase chain reaction, four (*MMP11*, *PLAU*, *BGN*, and *FAP*) are also present in our filtered data sets of significantly regulated probe sets comparing DCIS and invasive samples. They all show the same expression pattern as described by Schuetz and colleagues and are expressed at higher levels in the groups of invasive tumours. One of these genes (*MMP11*) is also part of the 35-gene and 80-gene classifiers. *MMP11 *and *PLAU *have already been correlated to invasion and poor prognosis [35,36]. *FAP *(seprase) is a membrane-bound protease that has been suggested to reduce the dependence of breast cancer cells on exogenous growth factors *in vitro *and thereby to facilitate tumour growth and metastasis [37]. Allinen and colleagues [38] identified comprehensive gene expression profiles of the different cell types in normal breast, DCIS, and invasive breast cancer tissue. These data show that dramatic gene expression changes occur between normal breast tissue and breast carcinomas and that these changes are already present at the DCIS stage. These results also suggest a role of the chemokines CXCL12 and CXCL14 in breast tumourigenesis. Neither chemokine is present on our array platform, but CXCR4, which is the receptor for CXCL12, is. *CXCR4 *does not appear in the set of significantly regulated genes, indicating that it does not play a crucial role in our series of tumours, which reflects the data of a mixed population of cells enriched for tumour cells, whereas Allinen and colleagues performed gene expression profiling on microdissected cell populations.

A recent study by Nagaraja and colleagues [39] describes gene expression patterns corresponding to normal breast, noninvasive breast cancer, and invasive breast cancer by using several cell lines. They identified genes involved in cell-cell and cell-matrix interactions which were altered in their expression. A set of nine genes was sufficient to distinguish between invasive and non-invasive cell lines [39]. From this set of nine transcripts, six could be matched to our array platform. For three of them (cadherin 11, annexin A1, and vimentin), we observe the same expression pattern as published by Nagaraja and colleagues for the transition from *in situ *to invasive carcinoma. The other three transcripts (S100A8, claudin 3, and cadherin 1) are upregulated in the invasive cancer cell line in the data set of Nagaraja and colleagues, whereas we see a downregulation in the invasive grade 3 tumours compared with the group of poorly differentiated samples. This may be due to the fact that Nagaraja and colleagues generated *in vitro *data, which we compared with our human breast cancer data set.

Porter and colleagues [40] identified a subset of genes that are significantly regulated in DCIS or invasive carcinomas. They identified 26 genes that were differentially expressed between normal and DCIS samples or intermediate- and high-grade DCIS, respectively. From these, only *XBP1 *is present in one of our classifiers (78 genes). Porter and colleagues describe this transcript as tumour-specific, meaning upregulated in *in situ *and invasive tumours compared with their normal samples. We find that *XBP1 *is significantly more highly expressed in well- and well-intermediately differentiated DCIS samples than in poorly/intermediately-poorly differentiated ones.

Wulfkuhle and colleagues [41] performed proteomic analyses of six matched normal and DCIS samples of the human breast. They identified proteins that are more highly expressed in individual DCIS samples and that are involved in cytoskeletal regulation or vesicular trafficking or have chaperone activity. From the 15 proteins from which the expression has been validated by IHC, 12 are present as probes on our array platform. Three of those (profilin, stathmin, and prohibitin) are differentially regulated between DCIS and invasive samples, and all three show a higher expression in the invasive samples than in the DCIS samples. This is in line with the paper of Wulfkuhle and colleagues, which describes a higher expression of these proteins in the DCIS samples than in normal tissue. This indicates that changes in gene and protein expression observed in invasive tumours are already present in the transition from normal tissue to DCIS lesions.

## Conclusion

We demonstrate here that gene expression profiling can distinguish between *in situ *breast cancer samples of well-versus poorly differentiated type. There appear to be a group of poorly differentiated samples, a group of well- and well-intermediately differentiated samples, and a third group containing mainly intermediately-poorly differentiated *in situ *cases. The quantitative differences in gene expression between these groups are mainly between twofold and fourfold. These differences are difficult to detect by classical IHC, because this technique is not very accurate in the quantification of small differences in protein expression. So far, there are no single markers that distinguish between the different types of DCIS, but the possibility of identifying a manageable panel of markers to distinguish the different types of DCIS lesions has to be further investigated.

## Abbreviations

DCIS = ductal carcinoma *in situ*; ER = oestrogen receptor; IDC = invasive ductal carcinoma; IHC = immunohistochemistry; LCIS = lobular carcinoma *in situ*; SNR = signal-to-noise ratio.

## Competing interests

The authors declare that they have no competing interests.

## Authors' contributions

JH performed data analyses, participated in the study design, and drafted the manuscript. AV participated in data analyses. JBGH and BK carried out microarray hybridisations. JP and MV reviewed the histological specimens. MV participated in designing the study and drafting the manuscript. All authors read and approved the final manuscript.

## Figures and Tables

**Table 1:** Patient characteristics

*In situ *samples		Invasive samples	
Differentiation	Number (percentage)	Histological grade	Number (percentage)

Well	6 (15%)	1	5 (12.5%)
Intermediately	18 (45%)	2	11 (27.5%)
Poorly	14 (35%)	3	24 (60%)
Good/poor component	1 (2.5%)		
LCIS	1 (2.5%)		
IHC		IHC	
ER-positive	28 (70%)^a^	ER-positive	22 (55%)^c^
PR-positive	24 (60%)^a^	PR-positive	19 (47.5%)^d^
Her2/neu-positive (3+)	12 (30%)^b^	Her2/neu-positive (3+)	4 (10%)^d^
p53-positive	11 (27.5%)^b^	p53-positive	9 (22.5%)^d^
Tumour detection			
Palpation	17 (42.5%)		
Microcalcifications	18 (45%)		
Others	5 (12.5%)		
Tumour diameter (mm)			
Range	10 to 80		
Median	45		
Average	42.8		
Treatment			
Mastectomy	24 (60%)		
Breast conserving treatment	6 (15%)		
Local excision followed by mastectomy	10 (25%)		

^a^5% not assessable, ^b^2.5% not assessable, ^c^27.5% not assessable, ^d^30% not assessable. ER, oestrogen receptor; IHC, immunohistochemistry; LCIS, lobular carcinoma *in situ*; PR, progesterone receptor.

**Table 2:** List of 35 genes able to discriminate between all DCIS and all invasive samples

Rank	NKI ID	Symbol	Annotation	Accession no.
1	116810	*ADM*	Adrenomedullin	AA446120
2	123346		EST	H17315
3	117289	*MMP11*	Matrix metalloproteinase 11 (stromelysin 3)	AA045500
4	121066	*DAPK3*	Death-associated protein kinase 3	AA973730
5	123776	*PIAS4*	Protein inhibitor of activated STAT protein	H30547
6	101837	*DHX34*	KIAA0134 gene product	AA477623
7	102847	*YIF1*	Putative transmembrane protein; homolog of yeast Golgi membrane protein Yif1p (Yip1p-interacting factor)	H79351
8	117345	*ACTN1*	Actinin, alpha 1	AA669042
9	127755	*TGFB2*	Transforming growth factor, beta 2	W47556
10	108960	*GABRD*	Gamma-aminobutyric acid (GABA) A receptor, delta	H41122
11	108348	*MFAP2*	Microfibrillar-associated protein 2	N67487
12	129658	*MGC13045*	DnaJ (Hsp40) homolog, subfamily C, member 4	AA996059
13	105479	*BAT3*	HLA-B-associated transcript-3	AA434416
14	120649	*KCTD5*	Hypothetical protein	AA521027
15	110728	*FBXL15*	F-box and leucine-rich repeat protein 15	T61547
16	120934	*EIF4G1*	Eukaryotic translation initiation factor 4 gamma, 1	R37276
17	118584	*C9orf115*	ESTs, weakly similar to B36298 proline-rich protein PRB3S [*Homo sapiens*]	AA479713
18	105533	*ARF1*	ADP-ribosylation factor 1	W45572
19	131909	*TUBB2*	Tubulin, beta polypeptide	AI672565
20	131540	*PRPF31*	DKFZP566J153 protein	AI253017
21	110281	*HSPA1L*	Heat shock 70-kD protein-like 1	H17513
22	107215	*KCTD5*	Hypothetical protein	AA429470
23	121937	*FLJ10374*	Hypothetical protein FLJ10374	AA676962
24	100368	*GNB2*	Guanine nucleotide binding protein (G protein), beta polypeptide 2	N68166
25	105453	*PSAP*	Prosaposin (variant Gaucher disease and variant metachromatic leukodystrophy)	N72215
26	115391	*LMCD1*	LIM and cysteine-rich domains 1	AA452125
27	128198	*MMP11*	Matrix metalloproteinase 11 (stromelysin 3)	AA954935
28	123688	*COL1A1*	Collagen, type I, alpha 1	R48844
29	127890	*PTMS*	Parathymosin	AA458981
30	102044	*DRAP1*	DR1-associated protein 1 (negative cofactor 2 alpha)	AA406285
31	101067	*MAP7*	Microtubule-associated protein 7	R77252 | R77251
32	129438	*IQGAP1*	IQ motif containing GTPase activating protein 1	AA478633
33	125700	*APC2*	Adenomatous polyposis coli like	AA976241
34	127881	*NFIC*	Nuclear factor I/C (CCAAT-binding transcription factor)	T59427
35	109065	*SYT5*	Synaptotagmin V	H39018

DCIS, ductal carcinoma *in situ*; EST, expressed sequence tag; NKI ID, Netherlands Cancer Institute (Amsterdam, The Netherlands) identification number.

**Table 3:** List of 80 genes able to discriminate between poorly differentiated DCIS and invasive grade 3 breast tumours

Rank	NKI ID	Symbol	Annotation	Accession no.
1	123776	*PIAS4*	Protein inhibitor of activated STAT protein	H30547
2	129658	*MGC13045*	DnaJ (Hsp40) homolog, subfamily C, member 4	AA996059
3	121937	*FLJ10374*	Hypothetical protein FLJ10374	AA676962
4	102847	*YIF1*	Putative transmembrane protein; homolog of yeast Golgi membrane protein Yif1p (Yip1p-interacting factor)	H79351
5	127755	*TGFB2*	Transforming growth factor, beta 2	W47556
6	117289	*MMP11*	Matrix metalloproteinase 11 (stromelysin 3)	AA045500
7	104973	*SYNPO2*	Synaptopodin 2	R31679
8	121066	*DAPK3*	Death-associated protein kinase 3	AA973730
9	128493	*GMFG*	Glia maturation factor, gamma	AI311932
10	105533	*ARF1*	ADP-ribosylation factor 1	W45572
11	132031		NY-REN-24 antigen	AA918005
12	127881	*NFIC*	Nuclear factor I/C (CCAAT-binding transcription factor)	T59427
13	120649	*KCTD5*	Potassium channel tetramerisation domain containing 5	AA521027
14	120934	*EIF4G1*	Eukaryotic translation initiation factor 4 gamma, 1	R37276
15	105453	*PSAP*	Prosaposin (variant Gaucher disease and variant metachromatic leukodystrophy)	N72215
16	112695	*SYNPO2*	*H. sapiens *cDNA FLJ20767 fis, clone COL06986	AA043349
17	101577	*BMI1*	Murine leukaemia viral (bmi-1) oncogene homolog	AA478036
18	105479	*BAT3*	HLA-B-associated transcript-3	AA434416
19	123071	*C9orf82*	Hypothetical protein FLJ13657	AA135972
20	101638	*ID4*	Inhibitor of DNA binding 4, dominant negative helix-loop-helix protein	AA464856
21	115306	*LRP16*	LRP16 protein	AA456318
22	118143	*STX1B2*	ESTs, moderately similar to ST1B_HUMAN SYNTAXIN 1B [*H. sapiens*]	H41572
23	128106	*DUSP6*	Dual specificity phosphatase 6	AA455254
24	115676	*RPS15A*	Ribosomal protein S15a	AA411682
25	108595	*CCL19*	Small inducible cytokine subfamily A (Cys-Cys), member 19	AA680186
26	126589	*C6orf166*	Hypothetical protein FLJ10342	AA984953
27	131540	*PRPF31*	DKFZP566J153 protein	AI253017
28	109065	*SYT5*		H39018
29	128198	*MMP11*	Matrix metalloproteinase 11 (stromelysin 3)	AA954935
30	109364	*MYST2*	Histone acetyltransferase	H11938
31	106989	*TNFSF13*	Tumour necrosis factor (ligand) superfamily, member 13	AA443577
32	109798			T82459
33	131890	*CDH1*	Cadherin 1, type 1, E-cadherin (epithelial)	AI671174
34	111513	*COG3*	*H. sapiens *clone 25226 mRNA sequence	AA461166
35	108645	*HMGCS2*	3-Hydroxy-3-methylglutaryl-Coenzyme A synthase 2 (mitochondrial)	AA496149
36	101651	*TRAP1*	Heat shock protein 75	AA497020
37	105304	*LRP16*	LRP16 protein	W52182 | AA284285
38	105363	*ARL7*	ADP-ribosylation factor-like 7	AA485683
39	127890	*PTMS*	Parathymosin	AA458981
40	118682	*NBS1*	Nijmegen breakage syndrome 1 (nibrin)	H98655
41	108997	*PTTG1IP*	Pituitary tumour-transforming 1 interacting protein	AA156461
42	110281	*HSPA1L*	Heat shock 70-kD protein-like 1	H17513
43	125700	*APC2*	Adenomatous polyposis coli like	AA976241
44	117139	*ALDOB*	Aldolase B, fructose-bisphosphate	H72098
45	107595	*SOX17*	SRY-box 17	AA427400 | AI732705
46	107375	*NUCKS*	Similar to rat nuclear ubiquitous casein kinase 2	AA137266
47	109238	*BSG*	Basigin (OK blood group)	AA436440
48	122821	*NSE2*	ESTs	H30453
49	123689	*LOC339123*	STIP1 homology and U-Box containing protein 1	R54844
50	115953	*LOC146542*	Human Chromosome 16 BAC clone CIT987SK-A-635H12	AA455010
51	108960	*GABRD*		H41122
52	128222	*GLUL*	Glutamate-ammonia ligase (glutamine synthase)	AI000103
53	100222	*NFIX*	Nuclear factor I/X (CCAAT-binding transcription factor)	AA406269
54	105470	*ISYNA1*	Myo-inositol 1-phosphate synthase A1	AA454554
55	117998	*RBM9*	RNA binding motif protein 9	H03903
56	105404	*GDF15*	Prostate differentiation factor	N26311
57	127811	*TOB1*	Transducer of ERBB2, 1	W96163
58	105524	*RPS6KA4*	Ribosomal protein S6 kinase, 90-kD, polypeptide 4	AA443601
59	109232	*BCKDHA*	Branched chain keto acid dehydrogenase E1, alpha polypeptide (maple syrup urine disease)	AA477298
60	115741	*APPL*	Adaptor protein containing pH domain, PTB domain and leucine zipper motif	AA436158
61	100898	*ELF3*	E74-like factor 3 (ets domain transcription factor, epithelial-specific)	AA434373
62	101067	*MAP7*	Microtubule-associated protein 7	R77252 | R77251
63	109306	*AQP1*	Aquaporin 1 (channel-forming integral protein, 28 kD)	H24316
64	102326	*CYC1*	Cytochrome c-1	AA447774
65	108988	*MALAT1*	Histone deacetylase 3	H88540
66	102253	*ACTG2*	Actin, gamma 2, smooth muscle, enteric	T60048
67	116834	*GPC1*	Glypican 1	AA455896
68	105497	*HNRPK*	Heterogeneous nuclear ribonucleoprotein K	W85697
69	108372	*LCP1*	Lymphocyte cytosolic protein 1 (L-plastin)	W73144
70	128634	*PRCP*	Prolylcarboxypeptidase (angiotensinase C)	AI360366
71	106297	*PHF17*	Hypothetical protein FLJ22479	AA136664
72	101616	*KRT19*	Keratin 19	AA464250
73	128532	*LTB*	Lymphotoxin beta (TNF superfamily, member 3)	AI351740
74	102385	*F13A1*	Coagulation factor XIII, A1 polypeptide	AA449742
75	102673	*WHSC1L1*	Wolf-Hirschhorn syndrome candidate 1-like 1	T97900
76	109638	*CXXC1*	CpG binding protein	T60082
77	109116	*FBL*	Fibrillarin	AA663986
78	109425	*TUBB*	Tubulin, beta polypeptide	AA427899
79	117500		EST	AA621138
80	100656	*UBE2C*	Ubiquitin carrier protein E2-C	AA430504

DCIS, ductal carcinoma *in situ*; EST, expressed sequence tag; NKI ID, Netherlands Cancer Institute (Amsterdam, The Netherlands) identification number.

**Table 4:** List of 43 genes able to discriminate between well- and poorly differentiated DCIS

Rank	NKI ID	Symbol	Annotation	Accession no.
1	108691	*ACK1*	Activated p21cdc42Hs kinase	AA427891
2	109246	*BCL2*	B-cell CLL/lymphoma 2	W63749
3	109268	*ALDH3A2*	Aldehyde dehydrogenase 3 family, member A2	AA633569
4	109236	*BTD*	Biotinidase	R17765
5	108595	*CCL19*	Small inducible cytokine subfamily A (Cys-Cys), member 19	AA680186
6	100524	*CELSR2*	Cadherin, EGF LAG seven-pass G-type receptor 2, flamingo (Drosophila) homolog	H39187
7	126868	*TMC4*	DKFZP586J0619 protein	AA991211
8	100708	*SLC39A6*	LIV-1 protein, oestrogen regulated	H29315
9	109170	*C4A*	Complement component 4A	AA664406
10	109127	*ESR1*	Oestrogen receptor 1	AA291749
11	128702		EST	AI313031
12	121012	*HSHIN1*	Hin-1	AA902831
13	128095	*PCSK6*	Paired basic amino acid cleaving system 4	W85807
14	128052	*ARHGEF7*	PAK-interacting exchange factor beta	AA452871
15	128493	*GMFG*	Glia maturation factor, gamma	AI311932
16	123382	*HIG1*	Likely ortholog of mouse hypoxia induced gene 1	T74105
17	129689	*C1orf21*	Chromosome 1 open reading frame 21	AA406569
18	102289	*ETFA*	Electron-transfer-flavoprotein, alpha polypeptide (glutaric aciduria II)	T57919
19	126124	*FLJ20152*	Hypothetical protein	AA918685
20	127815	*PLAT*	Plasminogen activator, tissue	R38933
21	101559	*NPY1R*	Neuropeptide Y receptor Y1	R43817
22	100260	*MAL*	Mal, T-cell differentiation protein	AA227885
23	127969	*CRYAA*	Crystallin, alpha A	H84722
24	128244	*SERPINA3*	Serine (or cysteine) proteinase inhibitor, clade A (alpha-1 antiproteinase, antitrypsin), member 3	AA704242
25	108649		Human clone 23948 mRNA sequence	H15114
26	106399	*GRTP1*	Hypothetical protein FLJ22474	N52651
27	123478	*FLJ14712*	Hypothetical protein FLJ14712	N79050
28	117207	*EMP3*	Epithelial membrane protein 3	W73810
29	111787	*ZNF451*	*H. sapiens *cDNA FLJ13010 fis, clone NT2RP3000542	AA486412
30	109502	*KITLG*	*H. sapiens *cDNA: FLJ21592 fis, clone COL07036	H11088
31	109315	*UCP2*	Uncoupling protein 2 (mitochondrial, proton carrier)	H61243
32	118532	*NUPL1*	PRO2463 protein	AA772502
33	100263	*MYB*	V-myb avian myeloblastosis viral oncogene homolog	N49284
34	128249	*CD3E*	CD3E antigen, epsilon polypeptide (TiT3 complex)	AA933862
35	131226	*IL7R*	Interleukin 7 receptor	T65739
36	100104	*SELL*	Selectin L (lymphocyte adhesion molecule 1)	H00662
37	108671	*BCAT2*	Branched chain aminotransferase 2, mitochondrial	AA436410
38	116984	*ATP5B*	ATP synthase, H+ transporting, mitochondrial F1 complex, beta polypeptide	AA708298
39	108376	*LAMA3*	Laminin, alpha 3 (nicein [150 kD], kalinin [165 kD], BM600 [150 kD], epilegrin)	AA001432
40	104944	*SLC7A2*	Solute carrier family 7 (cationic amino acid transporter, y+ system), member 2	R26163
41	100840	*THOC1*	Nuclear matrix protein p84	AA129297
42	100650	*SHFM1*	Deleted in split-hand/split-foot 1 region	H85464
43	101429	*SIAT1*	Sialyltransferase 1 (beta-galactoside alpha-2,6-sialytransferase)	AA598652

DCIS, ductal carcinoma *in situ*; EST, expressed sequence tag; NKI ID, Netherlands Cancer Institute (Amsterdam, The Netherlands) identification number.

**Table 5:** List of 78 genes able to discriminate between well/well-intermediately and intermediately-poorly/poorly differentiated DCIS

Rank	NKI ID	Symbol	Annotation	Accession no.
1	111275		EST	H20757
2	109268	*ALDH3A2*	Aldehyde dehydrogenase 3 family, member A2	AA633569
3	109236	*BTD*	Biotinidase	R17765
4	110384	*KPNA2*	Karyopherin alpha 2 (RAG cohort 1, importin alpha 1)	AA676460
5	131448	*PLEKHG1*	KIAA1209 protein	AI301815
6	108691	*ACK1*	Activated p21cdc42Hs kinase	AA427891
7	107840	*EPC1*	ESTs	AA120875
8	126868	*TMC4*	DKFZP586J0619 protein	AA991211
9	106257	*FLJ32499*	*H. sapiens *cDNA FLJ12749 fis, clone NT2RP2001149	W56590
10	128493	*GMFG*	Glia maturation factor, gamma	AI311932
11	128702			AI313031
12	129547	*METAP2*	Methionine aminopeptidase; eIF-2-associated p67	AA283030
13	111787	*ZNF451*	*H. sapiens *cDNA FLJ13010 fis, clone NT2RP3000542	AA486412
14	103209	*RBMS1*	*H. sapiens *mRNA; cDNA DKFZp564H0764 (from clone DKFZp564H0764)	R62566
15	108595	*CCL19*	Small inducible cytokine subfamily A (Cys-Cys), member 19	AA680186
16	129267			AA609203
17	109127	*ESR1*	Oestrogen receptor 1	AA291749
18	100263	*MYB*	V-myb avian myeloblastosis viral oncogene homolog	N49284
19	100524	*CELSR2*	Cadherin, EGF LAG seven-pass G-type receptor 2, flamingo (Drosophila) homolog	H39187
20	100260	*MAL*	Mal, T-cell differentiation protein	AA227885
21	102995	*PIGT*	CGI-06 protein	H82992
22	108649		Human clone 23948 mRNA sequence	H15114
23	109246	*BCL2*	B-cell CLL/lymphoma 2	W63749
24	100203	*TNFAIP3*	Tumour necrosis factor, alpha-induced protein 3	AA476272
25	107809	*XBP1*	X-box binding protein 1	W90128
26	102921		*H. sapiens *mRNA; cDNA DKFZp434D0818 (from clone DKFZp434D0818)	N95578
27	108671	*BCAT2*	Branched chain aminotransferase 2, mitochondrial	AA436410
28	101925	*EZH2*		AA430744
29	123382	*HIG1*	Likely ortholog of mouse hypoxia induced gene 1	T74105
30	131187	*KPNA2*	Karyopherin alpha 2 (RAG cohort 1, importin alpha 1)	AA489087
31	111288		*H. sapiens *mRNA; cDNA DKFZp564C2063 (from clone DKFZp564C2063)	AA416628
32	109170	*C4A*	Complement component 4A	AA664406
33	108203	*TEGT*	Testis enhanced gene transcript (BAX inhibitor 1)	AA629591
34	102639	*EML2*	Microtubule-associated protein like echinoderm EMAP	R27580
35	131258	*PSMA7*	Proteasome (prosome, macropain) subunit, alpha type, 7	AI318565
36	123478	*FLJ14712*	Hypothetical protein FLJ14712	N79050
37	109415	*FCGBP*	Fc fragment of IgG binding protein	R52030
38	127815	*PLAT*	Plasminogen activator, tissue	R38933
39	115769		ESTs	AA406313
40	106220	*GIMAP5*	Hypothetical protein FLJ11296	AA150443
41	128641	*PTTG1*	Pituitary tumour-transforming 1	AI362866
42	105439	*TGOLN2*	Trans-Golgi network protein (46-, 48-, 51-kD isoforms)	T81338
43	101362	*ERBB2*	V-erb-b2 avian erythroblastic leukaemia viral oncogene homolog 2 (neuro/glioblastoma derived oncogene homolog)	AA446928
44	108387	*IDH2*	Isocitrate dehydrogenase 2 (NADP+), mitochondrial	AA679907
45	100352	*TGOLN2*	Trans-Golgi network protein (46-, 48-, 51-kD isoforms)	H82891
46	107941	*PLAC8*	Hypothetical protein	AA150263
47	100104	*SELL*	Selectin L (lymphocyte adhesion molecule 1)	H00662
48	110983	*DLEU1*	Deleted in lymphocytic leukaemia, 1	AA425755
49	108438	*GRB7*	Growth factor receptor-bound protein 7	H53703
50	107752	*PAG*	Phosphoprotein associated with GEMs	N50114
51	128532	*LTB*	Lymphotoxin beta (TNF superfamily, member 3)	AI351740
52	124620	*ASTN2*	KIAA0634 protein	AA404602
53	102357	*CHN1*	Chimerin (chimaerin) 1	AA598668
54	109454	*AKR7A2*	Aldo-keto reductase family 7, member A2 (aflatoxin aldehyde reductase)	T62865
55	108678	*CASP10*	Caspase 10, apoptosis-related cysteine protease	H80712
56	131111	*CUGBP2*	CUG triplet repeat, RNA-binding protein 2	AA047257
57	123475	*C9orf87*	Hypothetical protein FLJ10493	N53432
58	105013		EST	H61003
59	100791	*TDG*	Thymine-DNA glycosylase	AA496947
60	100528	*BCL2L2*	BCL2-like 2	AA454588
61	116312	*FLJ14299*	Hypothetical protein FLJ14299	AA453170
62	100700	*TRIB2*	GS3955 protein	AA458653
63	102004	*PIK3R1*	Phosphoinositide-3-kinase, regulatory subunit, polypeptide 1 (p85 alpha)	R54050
64	104569	*MYO1B*	*H. sapiens *cDNA FLJ20153 fis, clone COL08656, highly similar to AJ001381 *H. sapiens *incomplete cDNA for a mutated allele	N95358
65	113907	*SNRPB2*	Small nuclear ribonucleoprotein polypeptide B"	H00286
66	128683	*WASL*	Wiskott-Aldrich syndrome-like	AI261600
67	123768	*DUSP22*	Mitogen-activated protein kinase phosphatase x	H42417
68	105099	*RET*	Ret proto-oncogene (multiple endocrine neoplasia MEN2A, MEN2B and medullary thyroid carcinoma 1, Hirschsprung disease)	H24956
69	116859	*STMN1*	Leukaemia-associated phosphoprotein p18 (stathmin)	AA873060
70	111660	*FLJ13710*	ESTs	AA120866
71	100112	*SAA1*	Serum amyloid A1	H25546
72	100840	*THOC1*	Nuclear matrix protein p84	AA129297
73	129239		EST, Moderately similar to AF119917 63 PRO2831 [*H. sapiens*]	W95750
74	115662	*GIMAP4*	Hypothetical protein FLJ11110	AA406363
75	109607	*HTPAP*	ESTs	T48412
76	108692	*EMP2*	Epithelial membrane protein 2	T88721
77	105133	*JUNB*	Jun B proto-oncogene	N94468
78	129959	*LOC283352*	EST	AI023540

DCIS, ductal carcinoma *in situ*; EST, expressed sequence tag; NKI ID, Netherlands Cancer Institute (Amsterdam, The Netherlands) identification number.
